# Leprosy: In Its Clinical & Pathological Aspects

**Published:** 1896-03

**Authors:** 


**Leprosy: in its Clinical & Pathological Aspects.** By Dr. G. Armauer Hansen and Dr. Carl Looft. Translated by Norman Walker, M.D. Pp. xi., 162. Bristol: John Wright & Co. 1895.

The clinical history of leprosy has been long and well written; but until recently no scientific description of its etiology and pathology could be held worthy of much estimation, because the discovery of the bacillus of leprosy, though made by Hansen as far back as 1871, was not clearly demonstrated until several years later, when improvements in the construction of immersion lenses left no doubt about the connection between the bacillus and the manifestation of this ancient and dire disease. Even to this time the demonstration is not scientifically proved; for there has been no successful inoculation of man and animals with the bacillus.

Having made these explanations, we have much pleasure in speaking in high terms of the work before us, which embodies the experience of two Norwegian physicians of large experience. The division of the disease into two main classes—the "nodular" and the "maculo-anæsthetic"—is simple and true. All cases come under one or both of these headings, the "leonine condition" being a high type of the first; and the neuritic cases, with their consequent motor and sensory pareses, good examples of the latter; and, between these, cases of an intermediate kind—the same disease, but of varying intensity according to the action of the bacilli. The "virulence of the bacilli seems to depend, not so much on any constant character of their own, as on the soil in which they live" (p. 79). One point is very clearly brought out; viz., that there is no such thing as leprosy of the lungs, intestines, bones, kidneys, and muscles. There may be secondary changes in these parts, and tubercular deposits in lungs and intestines, amyloid degeneration of kidneys and other viscera, and true deformities in joints and bones from muscular atrophy dependent upon neuritis—but not leprosy.

The chapter on Etiology is very interesting; and the question as to heredity of leprosy is fully dealt with, and disposed of negatively. In the town of Bergen the descendants of lepers may certainly be numbered by thousands, yet not one of them has developed the disease. The Leprosy Commission found only five or six per cent. of cases in the direct line; but children of perfectly healthy parents were leprous. Leprosy is undoubtedly contagious; but not hereditary. The chapter on treatment is fairly exhaustive, and shows how all the vaunted remedies, from flesh of crocodile and serpent, fat of lions and bears, to Koch's tuberculin, and all the oils pretty nearly in creation, are practically valueless. The consideration of this point is very important, because it enhances the value of the best known methods of treating this intractable disease; viz., by isolation and the enforcement of great cleanliness. Theoretically, the great desideratum of treatment would seem to be the destruction of the bacilli; but hitherto no remedies have done this. Chaulmoogra oil, pressed from the seeds of the gynocardia odorata, externally and internally applied, has been highly exalted; but looking over the records of a number of cases, it seems to us that the great cleanliness of the skin which is observed can well be credited with the good results which have attended some cases. "Later trials of the treatment in India have had the same negative results as in Norway" (p. 111).

In the work before us there is no allusion to the treatment suggested by Dr. Impey, of Robben Island. He has had large experience of the disease, and attaches great importance to the fact that certain febrile affections of the skin, *e.g.*, erysipelas and dermatitis, often caused the disappearance of the symptoms of leprosy, anyhow for a time; and he considers that the production of these affections, or attempts at it, by artificial means, might be productive of greater, if not permanent, benefits in the earlier stages of the complaint.

The tables towards the end of the volume, setting forth the salient points in cases of both forms of leprosy, the coincidence of tuberculosis, the ratio of the two forms of the disease, and the results of isolation in Norway, are well worth consulting; and the graphic plates of patients, the victims of leprosy, and of the bacilli and globi which are so characteristic, are exceedingly good, and some of them well coloured. The work is written clearly, and appears to us well translated. There is not an ambiguous sentence throughout; and anyone who wants information on the subject, cannot do better than consult the pages which Dr. Walker's care and energy have made acceptable.

Although the volume has an excellent and full table of contents, an index should also have been provided.

